# Supplementary material for: Do dogs rationally infer the causes of failed actions?
Source: PLoS One. 2026 Feb 13;21(2):e0341872. doi: 10.1371/journal.pone.0341872 (PMC12904391; doi:10.1371/journal.pone.0341872)
Supplement: S2 File — R markdown script for statistical analyses carried out for the study and reported in the results section of the manuscript. (PDF) [file pone.0341872.s002.pdf]

Do dogs rationally infer the causes of failed actions?

Amalia P. M. Bastos, Gavin R. Foster, Patrick M. Wood, Christopher Krupenye

This R Markdown file contains all analyses conducted on the data. Some analyses were pre-registered, and others were exploratory. These are noted throughout the document.

Before beginning our analyses, we download the required R packages.

```
library(readr)
library(tidyverse)
library(brms)
library(BayesFactor)
library(dplyr)
library(magrittr)
library(ggplot2)
```

Experiment 1 Analyses

First, we import our data into R. We set a seed value to improve reproducibility.

```
Exp1 <- read_csv('Gates_Exp1_Clean.csv')
set.seed("9192009")
```

Next, we convert "TRUE" and "FALSE" values to 0/1 binary values.

```
Exp1 <- Exp1 %>%
  mutate(Correct = case_when(
    Correct == TRUE ~ 1,
    Correct == FALSE ~ 0,
    is.na(Correct) ~ 0,
    TRUE ~ 0
  ))
```

Then, we calculate each dog's proportional success (i.e., in first approaching the competent agent) across their two trials.

```
Prop_Corrects <- Exp1 %>%
  group_by(Dog) %>%
  summarize(Success_Rate = mean(Correct))
```

We ensure that these proportions are listed in the correct order of dogs' participation in the study.

```
for(i in 1:nrow(Prop_Corrects)){
  Prop_Corrects$Order[i] <- Exp1$Order[which(Exp1$Dog==Prop_Corrects$Dog[i])[1]]
}
Prop_Corrects <- Prop_Corrects[order(Prop_Corrects$Order),]
```

Next, we perform Bayesian one-sample t-test with the current sample of dogs (N = 20), in line with our pre-registered stopping rules.

```
bf_result <- ttestBF(x = Prop_Corrects$Success_Rate,
                     mu = 0.5)

print(bf_result)
```

```
## Bayes factor analysis
## --------------
## [1] Alt., r=0.707 : 0.2888771 ±0.02%
##
## Against denominator:
##   Null, mu = 0.5
## ---
## Bayes factor type: BFoneSample, JZS
```

Given that the t-test indicates moderate support for the null hypothesis (BF < 0.3), we can now stop collecting data for this experiment and proceed onto the remaining analyses.

First, we use a multilevel logistic regression model to investigate whether dogs preferred to approach the competent agent first in each trial. This analysis was pre-registered.

```
Model_Exp1_Choice <- brm(Correct ~ (1|Dog),
                         data = Exp1,
                         save_pars = save_pars(all = TRUE),
                         family = bernoulli(link = "logit"),
                         iter = 12000,
                         warmup = 6000,
                         chains = 4,
                         cores = 4,
                         control = list(max_treedepth = 20))

summary(Model_Exp1_Choice)
```

```
##  Family: bernoulli
##   Links: mu = logit
## Formula: Correct ~ (1 | Dog)
##    Data: Exp1 (Number of observations: 40)
##   Draws: 4 chains, each with iter = 12000; warmup = 6000; thin = 1;
##          total post-warmup draws = 24000
##
## Multilevel Hyperparameters:
## ~Dog (Number of levels: 20)
##               Estimate Est.Error l-95% CI u-95% CI Rhat Bulk_ESS Tail_ESS
## sd(Intercept)     0.62      0.48     0.02     1.82 1.00     9981    12467
##
## Regression Coefficients:
##           Estimate Est.Error l-95% CI u-95% CI Rhat Bulk_ESS Tail_ESS
## Intercept     0.22      0.37    -0.49     0.98 1.00    22977    12156
##
## Draws were sampled using sampling(NUTS). For each parameter, Bulk_ESS
## and Tail_ESS are effective sample size measures, and Rhat is the potential
## scale reduction factor on split chains (at convergence, Rhat = 1).
```

Given that the confidence interval in the model above crosses zero, we find no support for the alternative hypothesis (that dogs prefer to approach the competent agent).

We compare this model to the null model below in order to obtain a Bayes factor for how strongly the data supports the alternative hypothesis.

```
Null_Model_Exp1_Choice <- brm(
  Correct ~ 1,
  data = Exp1,
  family = bernoulli(link = "logit"),
  iter = 12000,
  warmup = 6000,
  chains = 4,
  cores = 4,
  control = list(max_treedepth = 25, adapt_delta = 0.99)
)

bayes_factor(Model_Exp1_Choice, Null_Model_Exp1_Choice)
```

```
## Iteration: 1
## Iteration: 2
## Iteration: 3
## Iteration: 4
## Iteration: 5
## Iteration: 1
## Iteration: 2
## Iteration: 3
## Iteration: 4
```

```
## Estimated Bayes factor in favor of Model_Exp1_Choice over Null_Model_Exp1_Choice: 0.28240
```

This comparison shows that the data is moderately in favour of the null hypothesis (BF < 0.3). Therefore, in Experiment 1, dogs did not prefer to make their first approach to the competent agent rather than the incompetent agent.

As we mentioned in the pre-registration, we also attempted the same model accounting for trial order, with very similar results:

```
Model_Exp1_Choice_Trial <- brm(Correct ~ (1|Dog + Trial),
                              data = Exp1,
                              save_pars = save_pars(all = TRUE),
                              family = bernoulli(link = "logit"),
                              iter = 12000,
                              warmup = 6000,
                              chains = 4,
                              cores = 4,
                              control = list(max_treedepth = 25, adapt_delta = 0.99))

summary(Model_Exp1_Choice_Trial)
```

```
##  Family: bernoulli
##   Links: mu = logit
## Formula: Correct ~ (1 | Dog + Trial)
```

```
##    Data: Exp1 (Number of observations: 40)
##   Draws: 4 chains, each with iter = 12000; warmup = 6000; thin = 1;
##          total post-warmup draws = 24000
##
## Multilevel Hyperparameters:
## ~Dog (Number of levels: 20)
##               Estimate Est.Error l-95% CI u-95% CI Rhat Bulk_ESS Tail_ESS
## sd(Intercept)     0.67      0.53     0.03     1.96 1.00     9921    12491
##
## ~Trial (Number of levels: 2)
##               Estimate Est.Error l-95% CI u-95% CI Rhat Bulk_ESS Tail_ESS
## sd(Intercept)     1.29      1.25     0.05     4.58 1.00     9630    10566
##
## Regression Coefficients:
##           Estimate Est.Error l-95% CI u-95% CI Rhat Bulk_ESS Tail_ESS
## Intercept     0.19      0.98    -1.92     2.22 1.00    12448    11501
##
## Draws were sampled using sampling(NUTS). For each parameter, Bulk_ESS
## and Tail_ESS are effective sample size measures, and Rhat is the potential
## scale reduction factor on split chains (at convergence, Rhat = 1).
```

```
bayes_factor(Model_Exp1_Choice_Trial, Null_Model_Exp1_Choice)
```

```
## Iteration: 1
## Iteration: 2
## Iteration: 3
## Iteration: 4
## Iteration: 5
## Iteration: 1
## Iteration: 2
## Iteration: 3
## Iteration: 4
```

```
## Estimated Bayes factor in favor of Model_Exp1_Choice_Trial over Null_Model_Exp1_Choice: 0.15735
```

Next, for our first exploratory analysis, we calculate the total duration dogs spent at either gate for each row.

```
Exp1$Total_Duration <- Exp1$Duration_Competent + Exp1$Duration_Incompetent
```

We then use these values to calculate the proportion of time each dog spent at the gate near the competent agent, relative to the total time they spent in the vicinity of both gates combined.

```
Exp1$Proportion_Competent <- Exp1$Duration_Competent / Exp1$Total_Duration
```

Since some dogs would have spent no time at the gate closest to the competent agent, we assign very small values to these dogs so as to avoid an error for attempting to divide a value of 0. Therefore, we squeeze "Proportion_Competent" in case of values of exactly 0 or 1.

```
epsilon <- 1e-5
Exp1$Proportion_Competent <- pmin(pmax(Exp1$Proportion_Competent, epsilon), 1 - epsilon)
```

Now we can model whether dogs preferred to spend more time near the competent agent in each trial, as a proportion of the time they spent in the vicinity (defined as < 0.6m) of both gates. This is an exploratory analysis (not pre-registered).

```
Model_Exp1_Duration <- brm(Proportion_Competent ~ (1|Dog),
                                data = Exp1,
                                family = Beta(link = "logit"),
                                save_pars = save_pars(all = TRUE),
                                iter = 12000,
                                warmup = 6000,
                                chains = 4,
                                cores = 4,
                                control = list(max_treedepth = 20))

summary(Model_Exp1_Duration)
```

```
##  Family: beta
##   Links: mu = logit; phi = identity
## Formula: Proportion_Competent ~ (1 | Dog)
##    Data: Exp1 (Number of observations: 40)
##   Draws: 4 chains, each with iter = 12000; warmup = 6000; thin = 1;
##          total post-warmup draws = 24000
##
## Multilevel Hyperparameters:
## ~Dog (Number of levels: 20)
##               Estimate Est.Error l-95% CI u-95% CI Rhat Bulk_ESS Tail_ESS
## sd(Intercept)     0.18      0.15     0.01     0.54 1.00    14448    10613
##
## Regression Coefficients:
##           Estimate Est.Error l-95% CI u-95% CI Rhat Bulk_ESS Tail_ESS
## Intercept     0.07      0.19    -0.30     0.45 1.00    26804    17590
##
## Further Distributional Parameters:
##     Estimate Est.Error l-95% CI u-95% CI Rhat Bulk_ESS Tail_ESS
## phi     1.67      0.31     1.13     2.32 1.00    28350    16535
##
## Draws were sampled using sampling(NUTS). For each parameter, Bulk_ESS
## and Tail_ESS are effective sample size measures, and Rhat is the potential
## scale reduction factor on split chains (at convergence, Rhat = 1).
```

```
Null_Model_Exp1_Duration <- brm(Proportion_Competent ~ 1,
  data = Exp1,
  family = Beta(link = "logit"),
  iter = 12000,
  warmup = 6000,
  chains = 4,
  cores = 4,
  control = list(max_treedepth = 20)
)

bayes_factor(Model_Exp1_Duration, Null_Model_Exp1_Duration)
```

```
## Iteration: 1
```

```
## Iteration: 2
## Iteration: 3
## Iteration: 4
## Iteration: 5
## Iteration: 1
## Iteration: 2
## Iteration: 3
## Iteration: 4
```

```
## Estimated Bayes factor in favor of Model_Exp1_Duration over Null_Model_Exp1_Duration: 0.08173
```

We find strong evidence in favour of the null hypothesis (BF < 0.10), that is, that dogs did not spend a greater proportion of their time near the competent agent.

To visualise this result, we create a density plot showing the proportional amount of time spent at the gate near the competent agent, averaged across all dogs.

```
ggplot(Exp1, aes(x = Proportion_Competent)) +
  geom_density(alpha = 0.5, fill = "blue") +
  labs(title = "Density Plot of Proportional Time Spent Near Competent Agent Across All Dogs",
       x = "Proportion of Time Spent by Competent Agent",
       y = "Density") +
  theme_minimal() +
  theme(
    panel.grid.major = element_blank(),  # Remove major grid lines
    panel.grid.minor = element_blank()   # Remove minor grid lines
  )
```

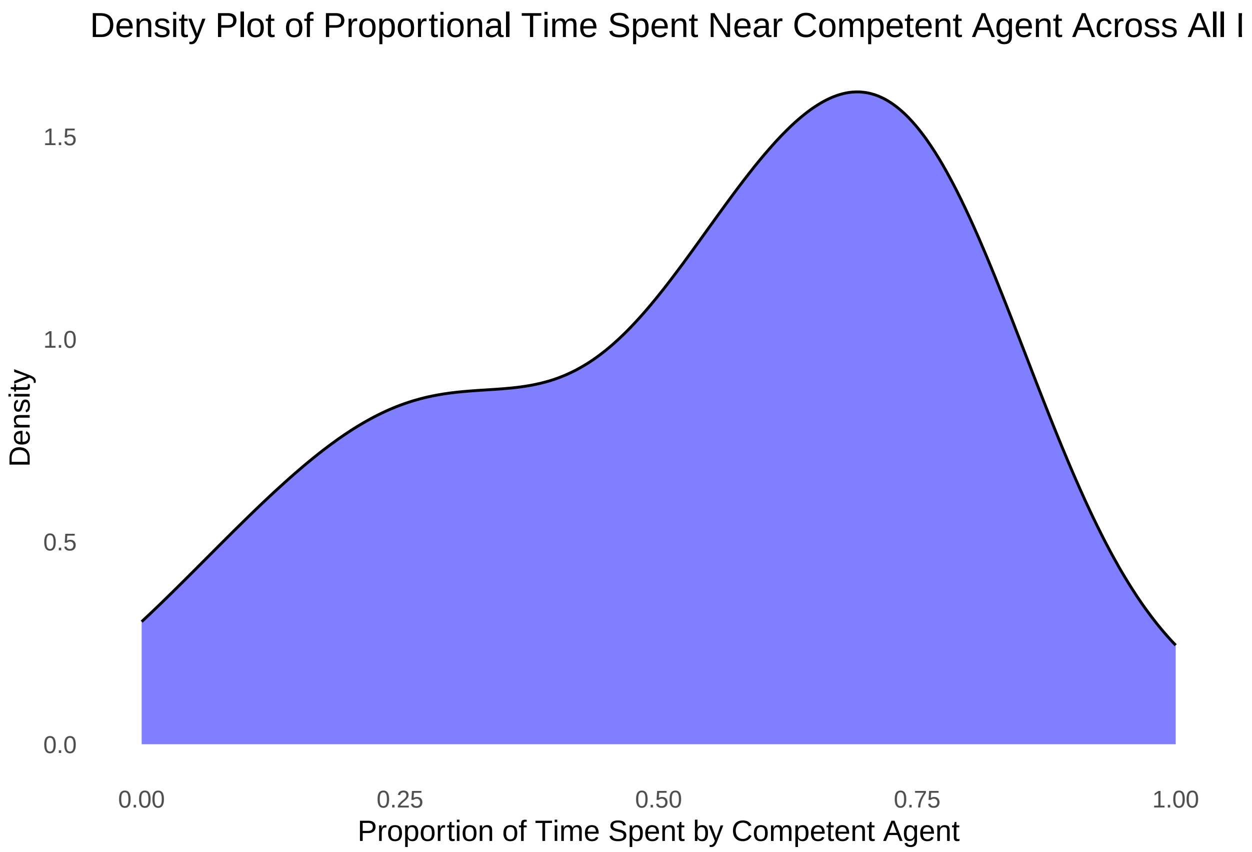

We also check for any possible effect of trial order for the same model:

```
Model_Exp1_Duration_Trial <- brm(Proportion_Competent ~ (1|Dog + Trial),
                                  data = Exp1,
                                  save_pars = save_pars(all = TRUE),
                                  family = Beta(link = "logit"),
                                  iter = 12000,
                                  warmup = 6000,
                                  chains = 4,
                                  cores = 4,
                                  control = list(max_treedepth = 25, adapt_delta = 0.99))

summary(Model_Exp1_Duration_Trial)
```

```
##  Family: beta
##   Links: mu = logit; phi = identity
## Formula: Proportion_Competent ~ (1 | Dog + Trial)
##    Data: Exp1 (Number of observations: 40)
##   Draws: 4 chains, each with iter = 12000; warmup = 6000; thin = 1;
##          total post-warmup draws = 24000
##
## Multilevel Hyperparameters:
## ~Dog (Number of levels: 20)
##               Estimate Est.Error l-95% CI u-95% CI Rhat Bulk_ESS Tail_ESS
## sd(Intercept)     0.19      0.15     0.01     0.55 1.00    17396    11362
##
## ~Trial (Number of levels: 2)
##               Estimate Est.Error l-95% CI u-95% CI Rhat Bulk_ESS Tail_ESS
## sd(Intercept)     1.03      1.24     0.03     4.27 1.00     3639     1561
##
## Regression Coefficients:
##           Estimate Est.Error l-95% CI u-95% CI Rhat Bulk_ESS Tail_ESS
## Intercept     0.06      0.82    -1.79     1.81 1.00     3741     1473
##
## Further Distributional Parameters:
##     Estimate Est.Error l-95% CI u-95% CI Rhat Bulk_ESS Tail_ESS
## phi     1.69      0.31     1.14     2.36 1.00    37060    16385
##
## Draws were sampled using sampling(NUTS). For each parameter, Bulk_ESS
## and Tail_ESS are effective sample size measures, and Rhat is the potential
## scale reduction factor on split chains (at convergence, Rhat = 1).
```

```
bayes_factor(Model_Exp1_Duration_Trial, Null_Model_Exp1_Duration)
```

```
## Iteration: 1
## Iteration: 2
## Iteration: 3
## Iteration: 4
## Iteration: 5
## Iteration: 6
## Iteration: 1
## Iteration: 2
## Iteration: 3
## Iteration: 4
```

```
## Estimated Bayes factor in favor of Model_Exp1_Duration_Trial over Null_Model_Exp1_Duration: 0.02472
```

Again, we find evidence for the null hypothesis when accounting for trial order (BF < 0.33).

Instead of attending to the competency of the two agents, it is possible that dogs instead followed some simpler behavioural heuristic, such as approaching the gate that was most recently opened. We used a Bayesian multilevel logistic regression to investigate this possibility. This was an exploratory (not pre-registered) analysis.

```
Model_Exp1_Opened <- brm(Choice_Side ~ Gate_Last_Opened + (1|Dog),
                                data = Exp1,
                                save_pars = save_pars(all = TRUE),
                                family = bernoulli(link = "logit"),
                                iter = 12000,
                                warmup = 6000,
                                chains = 4,
                                cores = 4,
                                control = list(max_treedepth = 20))

summary(Model_Exp1_Opened)
```

```
##  Family: bernoulli
##   Links: mu = logit
## Formula: Choice_Side ~ Gate_Last_Opened + (1 | Dog)
##    Data: Exp1 (Number of observations: 40)
##   Draws: 4 chains, each with iter = 12000; warmup = 6000; thin = 1;
##          total post-warmup draws = 24000
##
## Multilevel Hyperparameters:
## ~Dog (Number of levels: 20)
##               Estimate Est.Error l-95% CI u-95% CI Rhat Bulk_ESS Tail_ESS
## sd(Intercept)     1.47      0.98     0.08     3.77 1.00     5957    10210
##
## Regression Coefficients:
##                      Estimate Est.Error l-95% CI u-95% CI Rhat Bulk_ESS
## Intercept                1.12      0.70    -0.08     2.74 1.00    11159
## Gate_Last_OpenedRight   -0.77      0.98    -2.85     1.07 1.00    14882
##                      Tail_ESS
## Intercept                6597
## Gate_Last_OpenedRight    9992
##
## Draws were sampled using sampling(NUTS). For each parameter, Bulk_ESS
## and Tail_ESS are effective sample size measures, and Rhat is the potential
## scale reduction factor on split chains (at convergence, Rhat = 1).
```

```
Null_Model_Exp1_Opened <- brm(
  Choice_Side ~ 1,
  data = Exp1,
  family = bernoulli(link = "logit"),
  iter = 12000,
  warmup = 6000,
  chains = 4,
  cores = 4,
```

```r
  control = list(max_treedepth = 20)
)

bayes_factor(Model_Exp1_Opened, Null_Model_Exp1_Opened)
```

```
## Iteration: 1
## Iteration: 2
## Iteration: 3
## Iteration: 4
## Iteration: 5
## Iteration: 1
## Iteration: 2
## Iteration: 3
## Iteration: 4
```

```
## Estimated Bayes factor in favor of Model_Exp1_Opened over Null_Model_Exp1_Opened: 2.48560
```

We find no evidence to suggest that dogs preferred to approach the gate last opened by either agent.

Similarly, it is possible that dogs simply made their first approach to the gate most recently touched, regardless of whether it was successfully opened by an agent or not. Again, this was an exploratory analysis (not pre-registered).

```r
Model_Exp1_Touched <- brm(Choice_Side ~ Gate_Last_Touched + (1|Dog),
                              data = Exp1,
                              save_pars = save_pars(all = TRUE),
                              family = bernoulli(link = "logit"),
                              iter = 12000,
                              warmup = 6000,
                              chains = 4,
                              cores = 4,
                              control = list(max_treedepth = 20))

summary(Model_Exp1_Touched)
```

```
##  Family: bernoulli
##   Links: mu = logit
## Formula: Choice_Side ~ Gate_Last_Touched + (1 | Dog)
##    Data: Exp1 (Number of observations: 40)
##   Draws: 4 chains, each with iter = 12000; warmup = 6000; thin = 1;
##          total post-warmup draws = 24000
##
## Multilevel Hyperparameters:
## ~Dog (Number of levels: 20)
##               Estimate Est.Error l-95% CI u-95% CI Rhat Bulk_ESS Tail_ESS
## sd(Intercept)     1.39      0.92     0.08     3.60 1.00     5848     9165
##
## Regression Coefficients:
##                        Estimate Est.Error l-95% CI u-95% CI Rhat Bulk_ESS
## Intercept                  0.78      0.63    -0.34     2.17 1.00    16724
## Gate_Last_TouchedRight     0.12      0.85    -1.53     1.81 1.00    29745
##                        Tail_ESS
```

```
## Intercept                    11286
## Gate_Last_TouchedRight    16187
##
## Draws were sampled using sampling(NUTS). For each parameter, Bulk_ESS
## and Tail_ESS are effective sample size measures, and Rhat is the potential
## scale reduction factor on split chains (at convergence, Rhat = 1).
```

```
Null_Model_Exp1_Touched <- brm(
  Choice_Side ~ 1,
  data = Exp1,
  family = bernoulli(link = "logit"),
  iter = 12000,
  warmup = 6000,
  chains = 4,
  cores = 4,
  control = list(max_treedepth = 20)
)

bayes_factor(Model_Exp1_Touched, Null_Model_Exp1_Touched)
```

```
## Iteration: 1
## Iteration: 2
## Iteration: 3
## Iteration: 4
## Iteration: 5
## Iteration: 1
## Iteration: 2
## Iteration: 3
## Iteration: 4
```

```
## Estimated Bayes factor in favor of Model_Exp1_Touched over Null_Model_Exp1_Touched: 1.65503
```

We found no evidence to suggest that dogs preferentially approached the gate last touched.

Finally, we summarised the data to see if dogs exhibited a strong handedness bias - that is, to determine if dogs usually approached the gate on the same side of the room in both trials.

```
Handedness_Summary_Exp1 <- Exp1 %>%
  group_by(Dog) %>%
  summarize(Same_Choice = sum(Choice_Side[Trial == 1] == Choice_Side[Trial == 2]))

print(Handedness_Summary_Exp1)
```

```
## # A tibble: 20 x 2
##    Dog     Same_Choice
##    <chr>         <int>
##  1 Cully             0
##  2 Dutch             0
##  3 Gidget            1
##  4 Hadley            0
##  5 Hope              0
##  6 Hudson            1
##  7 Indy              0
```

```
##   8 Jax                 1
##   9 Kamden              0
##  10 Koji                1
##  11 Laney               0
##  12 Logan               1
##  13 Luna                1
##  14 Razor               1
##  15 Ripley              1
##  16 Sandy               1
##  17 Sawyer              1
##  18 Scout               0
##  19 Sophie              1
##  20 Watson              1
```

We find that 12 of 20 dogs chose the gate on the same side of the room in both trials, whilst 8 of 20 dogs changed which side of the room they first approached in each trial.

Experiment 2 Analyses

As before, we first import the data into R and set the same seed value.

```
Exp2 <- read_csv('Gates_Exp2_Clean.csv')
set.seed("9192009")
```

Then, we convert "TRUE" and "FALSE" values to 0/1 binary values.

```
Exp2 <- Exp2 %>%
  mutate(Correct = case_when(
    Correct == TRUE ~ 1,
    Correct == FALSE ~ 0,
    is.na(Correct) ~ 0,
    TRUE ~ 0
  ))
```

As before, we calculate each dog's proportional success (i.e., in first approaching the functional gate) across their two trials.

```
Prop_Corrects2 <- Exp2 %>%
  group_by(Dog) %>%
  summarize(Success_Rate = mean(Correct))
```

We ensure that these values are correctly listed according to the order dogs participated in the study.

```
for(i in 1:nrow(Prop_Corrects2)){
  Prop_Corrects2$Order[i] <- Exp2$Order[which(Exp2$Dog==Prop_Corrects2$Dog[i])[1]]
}
Prop_Corrects2 <- Prop_Corrects2[order(Prop_Corrects2$Order),]
```

Next, in line with our stopping rule, we carry out a Bayesian one-sample t-test at N = 20.

```r
bf_result <- ttestBF(x = Prop_Corrects2$Success_Rate,
                     mu = 0.5,
                     nullInterval = c(0, 1))

print(bf_result)
```

```
## Bayes factor analysis
## --------------
## [1] Alt., r=0.707 0<d<1    : 0.1956694 ±0%
## [2] Alt., r=0.707 !(0<d<1) : 0.519119  ±0%
##
## Against denominator:
##   Null, mu = 0.5
## ---
## Bayes factor type: BFoneSample, JZS
```

We find moderate support for the null hypothesis, that is, that dogs do not make first approaches to the functional gate more so than expected by chance (BF < 0.33). Since this is beyond the thresholds determined by our stopping rules (which require a BF > 3 or BF < 0.33), we do not need to collect further data.

As per our pre-registration, we investigate whether dogs preferred to approach the functional gate first in each trial using a Bayesian multilevel logistic regression:

```r
Model_Exp2_Choice <- brm(Correct ~ (1|Dog),
                              data = Exp2,
                              save_pars = save_pars(all = TRUE),
                              family = bernoulli(link = "logit"),
                              iter = 12000,
                              warmup = 6000,
                              chains = 4,
                              cores = 4,
                              control = list(max_treedepth = 20))

summary(Model_Exp2_Choice)
```

```
##  Family: bernoulli
##   Links: mu = logit
## Formula: Correct ~ (1 | Dog)
##    Data: Exp2 (Number of observations: 40)
##   Draws: 4 chains, each with iter = 12000; warmup = 6000; thin = 1;
##          total post-warmup draws = 24000
##
## Multilevel Hyperparameters:
## ~Dog (Number of levels: 20)
##               Estimate Est.Error l-95% CI u-95% CI Rhat Bulk_ESS Tail_ESS
## sd(Intercept)     1.41      0.93     0.09     3.61 1.00     5820     8539
##
## Regression Coefficients:
##           Estimate Est.Error l-95% CI u-95% CI Rhat Bulk_ESS Tail_ESS
## Intercept    -0.54      0.54    -1.70     0.43 1.00    11698     8390
##
## Draws were sampled using sampling(NUTS). For each parameter, Bulk_ESS
## and Tail_ESS are effective sample size measures, and Rhat is the potential
## scale reduction factor on split chains (at convergence, Rhat = 1).
```

The confidence intervals overlapping 0 suggest that this was not the case. We compare this model to a null model to obtain a Bayes Factor:

```
Null_Model_Exp2_Choice <- brm(
  Correct ~ 1,
  data = Exp2,
  family = bernoulli(link = "logit"),
  iter = 12000,
  warmup = 6000,
  chains = 4,
  cores = 4,
  control = list(max_treedepth = 20)
)

bayes_factor(Model_Exp2_Choice, Null_Model_Exp2_Choice)
```

```
## Iteration: 1
## Iteration: 2
## Iteration: 3
## Iteration: 4
## Iteration: 5
## Iteration: 1
## Iteration: 2
## Iteration: 3
## Iteration: 4
```

```
## Estimated Bayes factor in favor of Model_Exp2_Choice over Null_Model_Exp2_Choice: 0.95503
```

We find no support for the alternative hypothesis.

We also incorporate trial order into the model:

```
Model_Exp2_Choice_Trial <- brm(Correct ~ (1|Dog + Trial),
                                   data = Exp2,
                                   save_pars = save_pars(all = TRUE),
                                   family = bernoulli(link = "logit"),
                                   iter = 12000,
                                   warmup = 6000,
                                   chains = 4,
                                   cores = 4,
                                   control = list(max_treedepth = 25, adapt_delta = 0.99))

summary(Model_Exp2_Choice_Trial)
```

```
##  Family: bernoulli
##   Links: mu = logit
## Formula: Correct ~ (1 | Dog + Trial)
##    Data: Exp2 (Number of observations: 40)
##   Draws: 4 chains, each with iter = 12000; warmup = 6000; thin = 1;
##          total post-warmup draws = 24000
##
## Multilevel Hyperparameters:
## ~Dog (Number of levels: 20)
```

```
##                 Estimate Est.Error l-95% CI u-95% CI Rhat Bulk_ESS Tail_ESS
## sd(Intercept)     1.51      0.98     0.10     3.80 1.00     5996     9206
##
## ~Trial (Number of levels: 2)
##                 Estimate Est.Error l-95% CI u-95% CI Rhat Bulk_ESS Tail_ESS
## sd(Intercept)     1.16      1.23     0.03     4.50 1.00    10376    13252
##
## Regression Coefficients:
##           Estimate Est.Error l-95% CI u-95% CI Rhat Bulk_ESS Tail_ESS
## Intercept    -0.48      1.02    -2.50     1.68 1.00    12026    10765
##
## Draws were sampled using sampling(NUTS). For each parameter, Bulk_ESS
## and Tail_ESS are effective sample size measures, and Rhat is the potential
## scale reduction factor on split chains (at convergence, Rhat = 1).
```

```
bayes_factor(Model_Exp2_Choice_Trial, Null_Model_Exp2_Choice)
```

```
## Iteration: 1
## Iteration: 2
## Iteration: 3
## Iteration: 4
## Iteration: 1
## Iteration: 2
## Iteration: 3
## Iteration: 4
```

```
## Estimated Bayes factor in favor of Model_Exp2_Choice_Trial over Null_Model_Exp2_Choice: 0.37329
```

Again, we see no evidence to support the alternative hypothesis that dogs preferred to make first approaches to the functional gate compared to the non-functional gate.

In order to set up our first exploratory analysis, we calculate the total amount of time dogs spent in the vicinity (i.e., within 0.6m) of each of the two gates.

```
Exp2$Total_Duration <- Exp2$Duration_Functional + Exp2$Duration_Broken
```

Then, we use this to calculate the proportion of time each dog spent near the functional gate.

```
Exp2$Proportion_Functional <- Exp2$Duration_Functional / Exp2$Total_Duration
```

As in the Experiment 1 analysis, we now squeeze any values equivalent to 0 and 1.

```
epsilon <- 1e-5
Exp2$Proportion_Functional <- pmin(pmax(Exp2$Proportion_Functional, epsilon), 1 - epsilon)
```

In our first exploratory analysis for Experiment 2 data, we use a Bayesian multilevel logistic regression model to determine whether dogs preferred to spend a greater proportion of their time near the functional gate, relative to the total amount of time they spent in the vicinity of both gates.

```r
Model_Exp2_Duration <- brm(Proportion_Functional ~ (1|Dog),
                                data = Exp2,
                                save_pars = save_pars(all = TRUE),
                                family = Beta(link = "logit"),
                                iter = 12000,
                                warmup = 6000,
                                chains = 4,
                                cores = 4,
                                control = list(max_treedepth = 20))

summary(Model_Exp2_Duration)
```

```
##  Family: beta
##   Links: mu = logit; phi = identity
## Formula: Proportion_Functional ~ (1 | Dog)
##    Data: Exp2 (Number of observations: 40)
##   Draws: 4 chains, each with iter = 12000; warmup = 6000; thin = 1;
##          total post-warmup draws = 24000
##
## Multilevel Hyperparameters:
## ~Dog (Number of levels: 20)
##               Estimate Est.Error l-95% CI u-95% CI Rhat Bulk_ESS Tail_ESS
## sd(Intercept)     0.74      0.37     0.07     1.51 1.00     5480     7950
##
## Regression Coefficients:
##           Estimate Est.Error l-95% CI u-95% CI Rhat Bulk_ESS Tail_ESS
## Intercept    -0.18      0.27    -0.74     0.36 1.00    12741    12190
##
## Further Distributional Parameters:
##     Estimate Est.Error l-95% CI u-95% CI Rhat Bulk_ESS Tail_ESS
## phi     0.79      0.20     0.48     1.25 1.00     7748    13108
##
## Draws were sampled using sampling(NUTS). For each parameter, Bulk_ESS
## and Tail_ESS are effective sample size measures, and Rhat is the potential
## scale reduction factor on split chains (at convergence, Rhat = 1).
```

```r
Null_Model_Exp2_Duration <- brm(
  Proportion_Functional ~ 1,
  data = Exp2,
  family = Beta(link = "logit"),
  iter = 12000,
  warmup = 6000,
  chains = 4,
  cores = 4,
  control = list(max_treedepth = 20)
)

bayes_factor(Model_Exp2_Duration, Null_Model_Exp2_Duration)
```

```
## Iteration: 1
## Iteration: 2
## Iteration: 3
```

```
## Iteration: 4
## Iteration: 5
## Iteration: 1
## Iteration: 2
## Iteration: 3
## Iteration: 4

## Estimated Bayes factor in favor of Model_Exp2_Duration over Null_Model_Exp2_Duration: 0.70102
```

We find no evidence to support the alternative hypothesis.

We are also able to include trial order in the model to account for order effects:

```
Model_Exp2_Duration_Trial <- brm(Proportion_Functional ~ (1|Dog + Trial),
                                  data = Exp2,
                                  save_pars = save_pars(all = TRUE),
                                  family = Beta(link = "logit"),
                                  iter = 12000,
                                  warmup = 6000,
                                  chains = 4,
                                  cores = 4,
                                  control = list(max_treedepth = 20))

summary(Model_Exp2_Duration_Trial)
```

```
##  Family: beta
##   Links: mu = logit; phi = identity
## Formula: Proportion_Functional ~ (1 | Dog + Trial)
##    Data: Exp2 (Number of observations: 40)
##   Draws: 4 chains, each with iter = 12000; warmup = 6000; thin = 1;
##          total post-warmup draws = 24000
##
## Multilevel Hyperparameters:
## ~Dog (Number of levels: 20)
##               Estimate Est.Error l-95% CI u-95% CI Rhat Bulk_ESS Tail_ESS
## sd(Intercept)     0.73      0.37     0.07     1.49 1.00     4671     6582
##
## ~Trial (Number of levels: 2)
##               Estimate Est.Error l-95% CI u-95% CI Rhat Bulk_ESS Tail_ESS
## sd(Intercept)     0.94      1.06     0.02     3.83 1.00     5128     3915
##
## Regression Coefficients:
##           Estimate Est.Error l-95% CI u-95% CI Rhat Bulk_ESS Tail_ESS
## Intercept    -0.17      0.70    -1.72     1.40 1.00     4455     3106
##
## Further Distributional Parameters:
##     Estimate Est.Error l-95% CI u-95% CI Rhat Bulk_ESS Tail_ESS
## phi     0.79      0.20     0.48     1.25 1.00     6765    12632
##
## Draws were sampled using sampling(NUTS). For each parameter, Bulk_ESS
## and Tail_ESS are effective sample size measures, and Rhat is the potential
## scale reduction factor on split chains (at convergence, Rhat = 1).
```

```r
bayes_factor(Model_Exp2_Duration_Trial, Null_Model_Exp2_Duration)
```

```
## Iteration: 1
## Iteration: 2
## Iteration: 3
## Iteration: 4
## Iteration: 5
## Iteration: 1
## Iteration: 2
## Iteration: 3
## Iteration: 4
```

```
## Estimated Bayes factor in favor of Model_Exp2_Duration_Trial over Null_Model_Exp2_Duration: 0.20184
```

Again, we find no evidence to support the alternative hypothesis. The latter result offers moderate evidence in support of the null (BF < 0.33).

In order to visualise this data, we generate a density plot for the proportional amount of time dogs spent at the functional gate.

```r
ggplot(Exp2, aes(x = Proportion_Functional)) +
  geom_density(alpha = 0.5, fill = "blue") +
  labs(title = "Density Plot of Proportional Time Spent at Functional Gate Across All Dogs",
       x = "Proportion of Time Spent by Functional Gate",
       y = "Density") +
  theme_minimal() +
  theme(
    panel.grid.major = element_blank(),  # Remove major grid lines
    panel.grid.minor = element_blank()   # Remove minor grid lines
  )
```

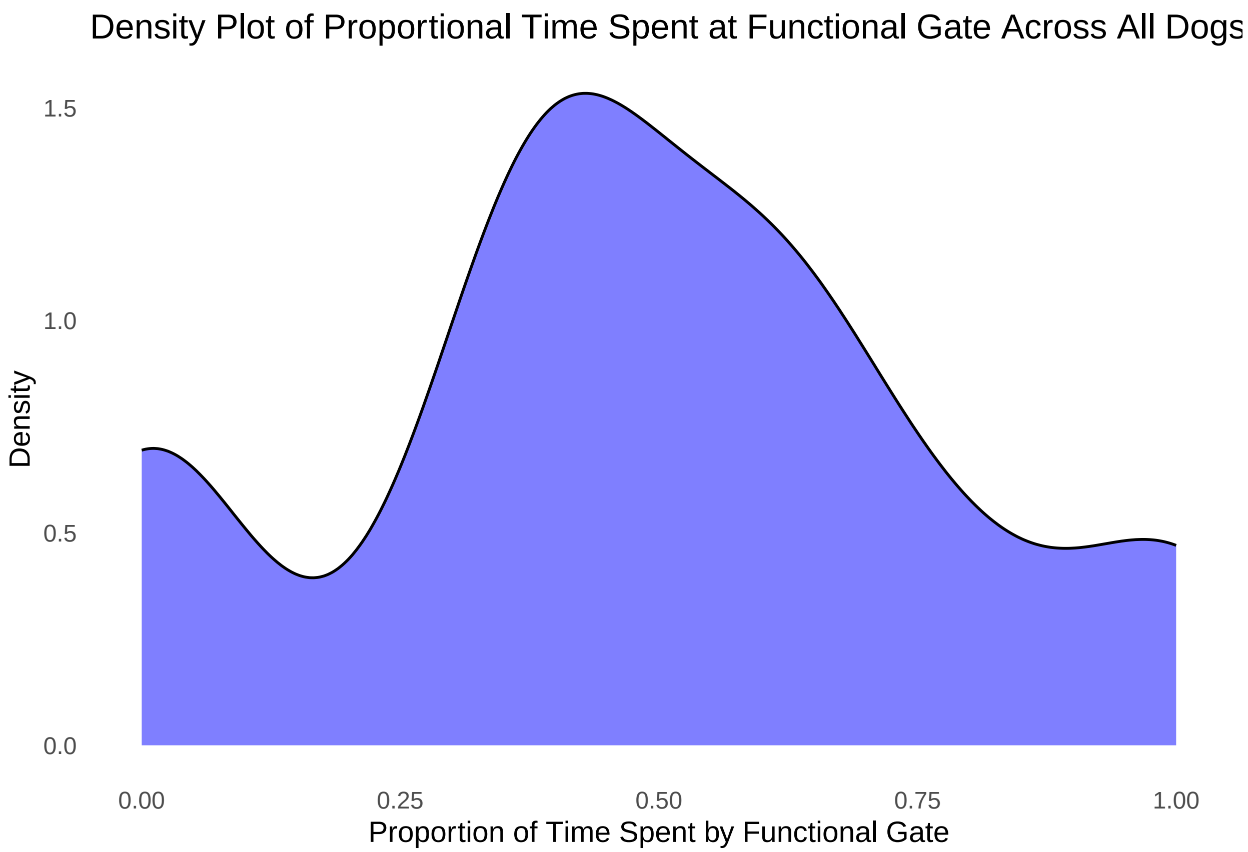

As before, it is possible that instead of attending to the functionality of the gates, dogs may have made their approach decisions based on simpler heuristics. One possible option is that dogs were simply drawn to approach the gate most recently touched. We used a Bayesian multilevel logistic regression for this exploratory analysis (which was not pre-registered).

```
Model_Exp2_Touched <- brm(Choice_Side ~ Gate_Last_Touched + (1|Dog),
                         data = Exp2,
                         save_pars = save_pars(all = TRUE),
                         family = bernoulli(link = "logit"),
                         iter = 12000,
                         warmup = 6000,
                         chains = 4,
                         cores = 4,
                         control = list(adapt_delta = 0.95, max_treedepth = 15))

summary(Model_Exp2_Touched)
```

```
##  Family: bernoulli
##   Links: mu = logit
## Formula: Choice_Side ~ Gate_Last_Touched + (1 | Dog)
##    Data: Exp2 (Number of observations: 40)
##   Draws: 4 chains, each with iter = 12000; warmup = 6000; thin = 1;
##          total post-warmup draws = 24000
##
## Multilevel Hyperparameters:
## ~Dog (Number of levels: 20)
```

```
##                Estimate Est.Error l-95% CI u-95% CI Rhat Bulk_ESS Tail_ESS
## sd(Intercept)     1.69      1.04     0.13     4.14 1.00     5448     8313
##
## Regression Coefficients:
##                         Estimate Est.Error l-95% CI u-95% CI Rhat Bulk_ESS
## Intercept                   0.13      0.70    -1.25     1.57 1.00    14484
## Gate_Last_TouchedRight     -0.92      0.88    -2.78     0.70 1.00    17894
##                         Tail_ESS
## Intercept                  13121
## Gate_Last_TouchedRight     13770
##
## Draws were sampled using sampling(NUTS). For each parameter, Bulk_ESS
## and Tail_ESS are effective sample size measures, and Rhat is the potential
## scale reduction factor on split chains (at convergence, Rhat = 1).
```

```
Null_Model_Exp2_Touched <- brm(
  Choice_Side ~ 1,
  data = Exp2,
  family = bernoulli(link = "logit"),
  iter = 12000,
  warmup = 6000,
  chains = 4,
  cores = 4,
  control = list(max_treedepth = 20)
)

bayes_factor(Model_Exp2_Touched, Null_Model_Exp2_Touched)
```

```
## Iteration: 1
## Iteration: 2
## Iteration: 3
## Iteration: 4
## Iteration: 5
## Iteration: 6
## Iteration: 1
## Iteration: 2
## Iteration: 3
```

```
## Estimated Bayes factor in favor of Model_Exp2_Touched over Null_Model_Exp2_Touched: 4.08256
```

Although variability across subjects was large, we find evidence that the model including last touched gate is moderately better at explaining first approach data compared to the null model (BF > 3).

Finally, we again summarised the data to determine if dogs tended to approach the same side on both trials, which might indicate that dogs exhibit strong side biases in this task (note that the side of the “functional” gate was swapped between trials within each dog).

```
Handedness_Summary_Exp2 <- Exp2 %>%
  group_by(Dog) %>%
  summarize(Same_Choice = sum(Choice_Side[Trial == 1] == Choice_Side[Trial == 2]))

print(Handedness_Summary_Exp2)
```

```
## # A tibble: 20 x 2
##    Dog       Same_Choice
##    <chr>           <int>
##  1 Beatrice            1
##  2 Bonnie              0
##  3 Buster              1
##  4 Carly               0
##  5 Ella                0
##  6 Kota                1
##  7 Kudos               1
##  8 Mabel               1
##  9 Marble              0
## 10 Miller              0
## 11 Nyx                 1
## 12 Odin                1
## 13 Piper               0
## 14 Renny               1
## 15 Rosie               1
## 16 Rosita              0
## 17 Storm               0
## 18 Tela                0
## 19 Tess                1
## 20 Tucker              1
```

We find that 11 of 20 dogs approached the same side first, suggesting that dogs did not overwhelmingly side bias at the group level.
